# The Binding Mechanisms of Antibodies to DNA from Healthy Subjects and Patients with Systemic Lupus Erythematosus: The Role of Monogamous Bivalency and Fc Dependence

**DOI:** 10.4049/immunohorizons.2100077

**Published:** 2021-10-08

**Authors:** Morgan E. Belina, Diane M. Spencer, David S. Pisetsky

**Affiliations:** * Duke University School of Medicine, Duke University Medical Center, Durham, NC; † Division of Rheumatology and Immunology, Duke University Medical Center, Durham, NC; ‡ Medical Research Service, Durham Veterans Administration Medical Center, Durham, NC

## Abstract

Abs to DNA (anti-DNA) are a unique population of Abs that bind structural determinants on the DNA molecule. In systemic lupus erythematosus (SLE), anti-DNA Abs bind to conserved antigenic determinants, with the phosphodiester backbone being the most likely. In contrast, otherwise healthy subjects (HS) express anti-DNA that bind selectively to nonconserved sites on certain bacterial and viral DNA. As shown previously, SLE anti-DNA bind by a mechanism termed Fc-dependent monogamous bivalency. In this mechanism, both Fab sites interact with determinants on the same extended DNA molecule, reflecting the low affinity of each Fab site; the requirement for the Fc region suggests some contribution of the C region to increase avidity. In this study, we investigated whether anti-DNA from HS also bind to bacterial DNA by Fc-dependent monogamous bivalency. For this purpose, we compared the activity of intact IgG with Fab and F(ab′)_2_ fragments prepared from the plasmas of SLE patients and HS using ELISAs with DNA from calf thymus or *Micrococcus luteus*. These studies showed that Fab fragments from all plasmas tested, both SLE and HS, failed to bind significantly to DNA compared with intact IgG. By contrast, some, but not all, F(ab′)_2_ preparations from both SLE patients and HS showed binding to *M. luteus* DNA; F(ab′)_2_ fragments from SLE plasmas, however, did not bind significantly to calf thymus DNA. Together, these findings suggest that although anti-DNA Abs, whether from SLE or HS, bind by monogamous bivalency, binding to bacterial DNA does not require the Fc region.

## INTRODUCTION

Abs to DNA (anti-DNA) are a unique population of Abs that bind structural determinants on the DNA molecule (1, 2). These Abs were originally defined in the context of systemic lupus erythematosus (SLE), a prototypic autoimmune disease characterized by the production of autoantibodies to nuclear macromolecules (3–6). The serological hallmark of SLE, anti-DNA are important biomarkers for disease diagnosis, classification, and activity (7). Furthermore, anti-DNA are important mediators of disease manifestations via the formation of immune complexes (8). The ability of anti-DNA in SLE to bind DNA independent of species origin has suggested recognition of widely shared or conserved antigenic determinants, with the phosphodiester backbone being the most likely (9, 10).

The role of anti-DNA in SLE diagnosis and classification has suggested that the production of IgG anti-DNA is exclusive to the autoimmune state. However, data assembled over the last decades have clearly demonstrated the presence of IgG Abs in the blood of otherwise healthy subjects (HS) to DNA from certain bacteria and viruses (2, 11–14); these IgG Abs differ from low-affinity IgM, so-called natural autoantibodies that can bind DNA (15). Levels of Abs to bacterial DNA in the blood of HS can be comparable to those of autoantibodies to DNA in patients with SLE, indicating a robust immune response (11).

Although the origin of anti-DNA in general has not been delineated, Abs to foreign DNA likely arise in the context of infection or colonization. As now recognized, the microbiome can exert powerful effects on the immune system in both HS and patients with SLE; DNA from biofilms in particular has been shown to be immunogenic (16–18). Furthermore, studies on circulating cell-free DNA (cfDNA) indicate that cfDNA from bacteria and fungi, among other species, is present in the blood of infected individuals (19–21). Together, these findings suggest that nucleic acids can interact with the immune system at sites distinct from any localized sources of infection such as a pneumonia or abscess. The production of Abs to foreign DNA may be one consequence of such interactions.

As our studies have demonstrated, the anti-DNA in HS, unlike autoantibodies to DNA in SLE, are highly selective in their pattern of Ag interaction and target nonconserved sites distinct from the phosphodiester backbone (22). Anti-DNA in HS also differ from SLE anti-DNA in their pattern of H and L chain expression (23). In recent studies, we explored the nature of autoantibody-DNA interactions in SLE, delineating the role of monogamous bivalency in anti-DNA binding (24). In monogamous bivalency, both Fab regions of an IgG molecule must contact sites along the same extended polynucleotide chain for stable interaction; the binding of each Fab alone is too weak to allow univalent binding (25–27). This mode of binding requires a piece of DNA with a minimum size of ~35–40 bp, which is sufficiently long to span the ~140-Å distance between the two Fab sites on an intact IgG molecule (26, 28).

As expected, we showed that Fab fragments of IgG purified from the blood of patients with SLE failed to bind DNA in an ELISA using calf thymus (CT) DNA as the source of mammalian dsDNA (24). Our work produced an additional, surprising result: we found that F(ab′)_2_ fragments of SLE IgG failed to bind DNA, even though such fragments should be capable of a monogamous bivalent interaction. Previous studies have demonstrated the importance of the Fc region to the binding of anti-DNA and other Ags, although such studies involved molecular constructs with the same variable regions joined to different IgG H chains (29–31). In our studies, by contrast, we used F(ab′)_2_ fragments lacking an Fc region. To describe the lack of both Fab fragment and F(ab′)_2_ fragment reactivity to native DNA in SLE, we termed this pattern of anti-DNA binding as Fc-dependent monogamous bivalency (24).

In this study, we further investigated the binding of HS anti-DNA to bacterial DNA. Specifically, we produced Fab and F(ab′)_2_ fragments of IgG from the plasmas of HS to test whether HS anti-DNA display monogamous bivalency and, if so, whether such bivalency is Fc dependent. In addition, we compared the binding activities of HS anti-DNA and SLE anti-DNA with DNA from *Micrococcus luteus*, previously known as *M. lysodeikticus*. In the results presented in this study, we show that Fab fragments of HS IgG, like those from SLE IgG, fail to bind to *M. luteus* DNA. In contrast, we demonstrate that F(ab′)_2_ fragments from some, but not all, HS and SLE plasmas are able to bind *M. luteus* DNA. These findings thus extend the role of monogamous bivalency to HS Abs to bacterial DNA while indicating some capacity for Fc independence in the IgG Ab response to bacterial DNA in both HS and patients with SLE.

## MATERIALS AND METHODS

### Source of reagents

All chemicals were purchased from Sigma-Aldrich (St. Louis, MO), unless otherwise noted. Non-SLE plasmas were obtained from Innovative Research (Novi, MI); SLE plasmas were obtained from Plasma Services Group (Huntingdon Valley, PA). EBV Ag was from Meridian Life Science (Memphis, TN). PBS (Ca^2+^ and Mg^2+^ free) and saline sodium citrate solution were purchased from Invitrogen, Thermo Fisher Scientific (Waltham, MA).

### Isolation of IgG

Purification of IgG from plasmas as well as a pooled, technical grade human IgG preparation was accomplished using 0.2 ml NAb Protein A/G Spin Columns (Thermo Fisher Scientific/Pierce Biotechnology, Rockford, IL) following the supplier’s protocol. Binding buffer used was 0.01 M sodium phosphate and 0.15 M sodium chloride (pH 7.2); elution buffer used was 0.1 M glycine (pH 2.5). Eluted fractions of IgG were neutralized by addition of 1/10 volume of neutralization buffer (1 M Tris HCl [pH 8.9]). Concentrations of IgG were determined from measurements of A280 with a Thermo Scientific NanoDrop 1000 spectrophotometer, read in undiluted form; blanking solution was chosen to be as similar as possible to the solvent of IgG samples. IgG preparations were stored at 4°C until use.

### Generation of Fab fragments

Monovalent Fab fragments were prepared from purified IgG by papain digestion using the Pierce Fab Micro Preparation Kit (Thermo Fisher Scientific/Pierce Biotechnology) according to the supplier’s protocol as follows. IgG was diluted to 2 mg/ml and buffer exchanged to digestion buffer (supplied proprietary buffer with cysteine added to 20 mM) with desalting columns. IgG was then incubated with immobilized papain at 37°C for 5 h with end-over-end rotation. The digest was removed from the immobilized papain by centrifugation; Fab fragments were isolated using a protein A spin column. Fc fragments and undigested IgG were eluted from the protein A spin column with elution buffer (supplied proprietary buffer [pH 2.8], containing primary amine) and neutralized by addition of 1/10 volume of neutralization buffer (1 M Tris HCl [pH 8.9]). To assess the completeness of the digestion, Fab fragments, and undigested IgG and Fc fragments were evaluated via nonreducing SDS-PAGE using NuPAGE 4–12% Bis Tris gels (Invitrogen, Thermo Fisher Scientific). Samples were mixed with LDS sample buffer (Invitrogen, Thermo Fisher Scientific) and heated for 10 min at 70°C before loading. Fab fragments were quantitated and stored using the methods outlined above.

### Generation of F(ab′)_2_ fragments

F(ab′)_2_ fragments were prepared from purified IgG by pepsin digestion using the Pierce F(ab′)_2_ Micro Preparation Kit (Thermo Fisher Scientific/Pierce Biotechnology) according to the supplier’s protocol as follows. IgG was diluted to 2 mg/ml and buffer exchanged to digestion buffer (20 mM sodium acetate [pH 4.4] and 0.05% sodium azide) with desalting columns. IgG was then incubated with immobilized pepsin at 37°C for 2 h with end-over-end rotation. The digest was removed from the immobilized pepsin by centrifugation; F(ab′)_2_ fragments were isolated using a protein A spin column. Fc fragments and undigested IgG were eluted from the protein A spin column with elution buffer (supplied proprietary buffer [pH 2.8], containing primary amine) and neutralized by addition of 1/10 volume of neutralization buffer (1 M Tris HCl [pH 8.9]). To assess digestion completion, fractions containing pepsin digest, F(ab′)_2_ fragments, and undigested IgG and Fc fragments were evaluated via nonreducing SDS-PAGE. Samples were mixed with LDS sample buffer (Invitrogen, Thermo Fisher Scientific) and heated for 10 min at 70°C before loading. F(ab′)_2_ fragments were quantitated and stored using the methods outlined above.

### Calculation of concentrations of IgG, Fab, and F(ab′)_2_ for use in ELISAs

As the purpose of these experiments was to assess the relative binding activity of intact IgG, Fab fragments, and F(ab′)_2_ fragments, the initial concentration of each reagent was calculated to provide equivalent numbers of Ag-binding sites. The molecular mass of an intact IgG molecule was assumed to be 160 kDa and the molecular mass of one Fab fragment to be 50 kDa. The weight of a preparation of Fab fragments containing the same number of Ag-binding sites as an intact IgG molecule (2) was calculated as two Fab fragments × 50 kDa, hence 100 kDa. Therefore, for example, the binding activity of 20 μg/ml intact IgG was compared with the binding activity of 12.5 μg/ml Fab fragment preparation (calculated as 20 μg/ml × 100 kDa/160 kDa) in the experiments of this study.

Each F(ab′)_2_ fragment theoretically contains the same number of Ag-binding sites as an intact IgG molecule. Preparations of F(ab′)_2_ contained not only F(ab′)_2_ fragments but also Fc fragments remaining after pepsin digestion. Assuming that the concentration of Fc fragments remaining in F(ab′)_2_ preparations was not negligible, the weight of a preparation of F(ab′)_2_ fragments could be concluded to have an identical number of Ag-binding sites as the same weight of intact IgG (24). Thus, for example, the binding activity of 20 μg/ml intact IgG was compared with the binding activity of 20 μg/ml F(ab′)_2_ fragment preparation.

### ELISAs

ELISAs were conducted as previously described (24). Briefly, high-binding, flat-bottom, 96-well microtiter plates (Immulon 2HB; Thermo Fisher Scientific) were used. All washes were performed three times with 1× PBS. All reagent volumes were 100 μl/well and all incubations at room temperature (21–23°C) for 1 h except when otherwise noted. Secondary Ab used was anti-human IgG (Fab specific)–peroxidase diluted 1/300 in 50 mM Tris (pH 7.4) containing 0.1% BSA and 0.05% Tween 20 (ELISA Dilution Buffer).

Wells of plates were coated with 5 μg/ml *M. luteus* or CT DNA diluted in 1× SSC buffer, 1 μg/ml tetanus toxoid diluted in ELISA coating buffer (0.1 M sodium phosphate buffer [pH 9]), or 6.6 μg/ml EBV Ag diluted in 1× PBS and incubated overnight at 4°C. Plates were then washed and blocked with 200 μl/well PBS containing 2% BSA and 0.05% Tween 20 (Block Buffer II) for 2 h. Plates were washed, and wells were incubated with serial 2-fold dilutions of IgG, Fab, or F(ab′)_2_ fragments in ELISA Dilution Buffer. Plates were then washed, and wells were incubated with secondary Ab. Finally, plates were washed and incubated with HRP substrate (0.015% 3′3′,5,5′-tetramethylbenzidine dihydrochloride, 0.01% H_2_O_2_ in 0.1 M citrate buffer [pH 4]) for 30 min. Color development was halted with addition of 2 M H_2_SO_4_, and the absorbance of wells at 450 nm was measured with a UV_max_ spectrophotometer (Molecular Devices, San Jose, CA).

## RESULTS

### The binding of IgG, Fab, and F(ab′)_2_ fragments from HS

Previous studies have indicated that SLE anti-DNA bind to DNA by a mechanism termed monogamous bivalency, in which both Fab sites must contact antigenic determinants on the same polynucleotide chain (24, 26). In other studies, we showed that F(ab′)_2_ fragments of SLE IgG, like Fab fragments, failed to bind mammalian DNA even though these fragments should be capable of monogamous bivalency. We designated this mode of binding as Fc-dependent monogamous bivalency (24). We thus wished to determine whether HS anti-DNA also bind by this mechanism in their interaction with bacterial DNA.

**FIGURE 1.**
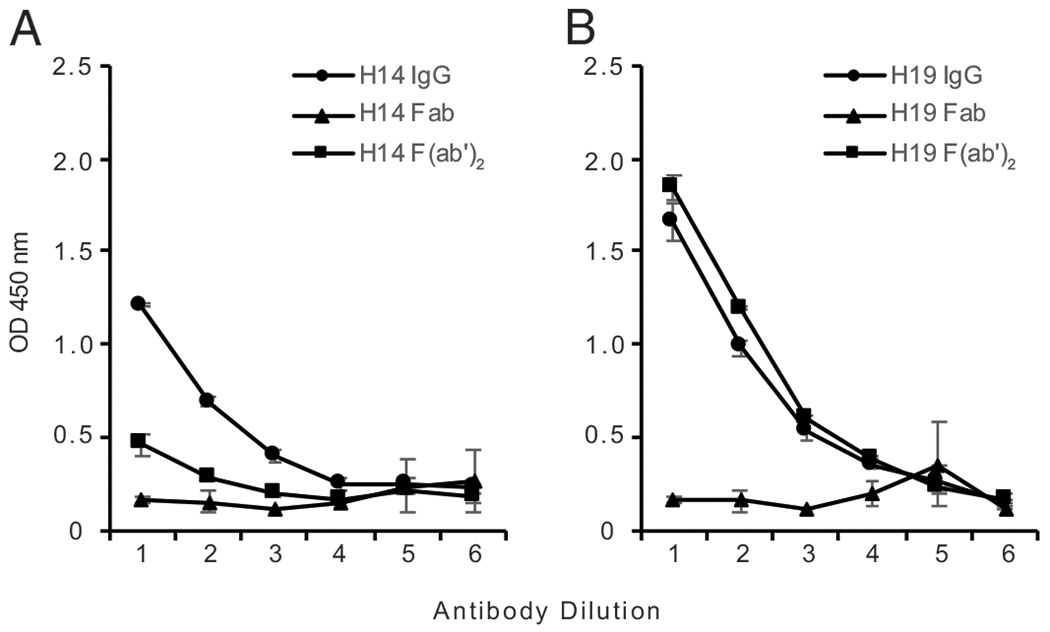
Binding of representative HS IgG, Fab, and F(ab′)_2_ fragment preparations to *M*. luteus DNA. The binding of IgG (circle), Fab (triangle), and F(ab′)_2_ (square) fragments from two representative HS plasmas (H14 and H19) to *M. luteus* DNA was examined by ELISA. Each point shown is the average OD_450_ of two wells, with error bars indicating SD. Serial 2-fold dilutions of IgG and fragments were tested, with initial concentrations as follows. For plasma H14 (**A**): IgG and F(ab′)_2_, 20 μg/ml; Fab, 12.5 μg/ml. For plasma H19 (**B**): IgG and F(ab′)_2_, 80 μg/ml; Fab, 50 μg/ml.

**FIGURE 2.**
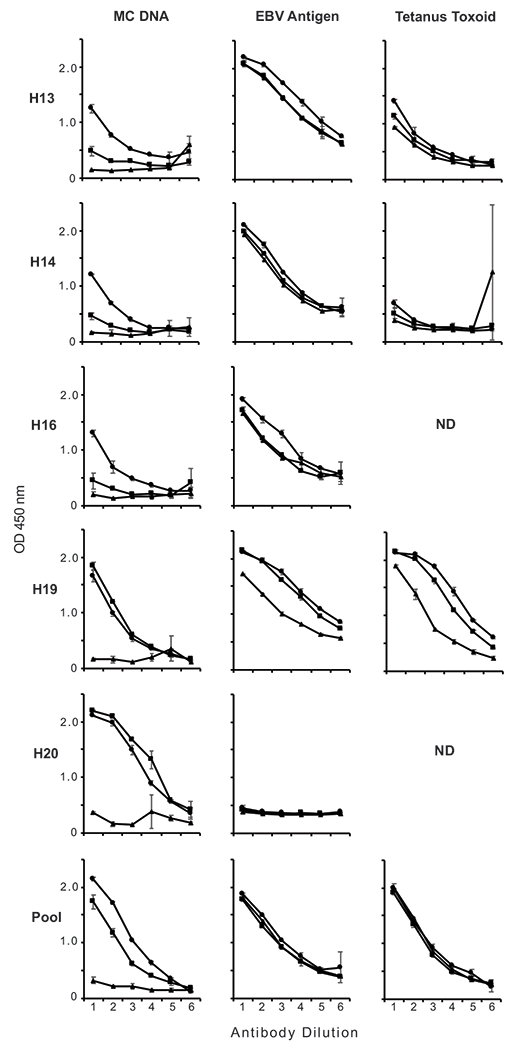
Binding of HS IgG, Fab, and F(ab′)_2_ fragment preparations to *M*. luteus DNA and controls. The binding of IgG (circle), Fab (triangle), and F(ab′)_2_ (square) fragments from five HS plasmas (H13, H14, H16, H19, and H20) and preisolated, pooled IgG (Pool) to *M. luteus* DNA, EBV Ag, and tetanus toxoid was examined by ELISA. ND, data not determined for a particular control. Each point shown is the average OD_450_ of two wells, with error bars indicating SD. Serial 2-fold dilutions of IgG and fragments were tested, with initial concentrations as follows. For plasmas H13, H14, H16, H20, and Pool: IgG and F(ab′)_2_, 20 μg/ml; Fab, 12.5 μg/ml. For plasma H19: IgG and F(ab′)_2_, 80 μg/ml; Fab, 50 μg/ml.

In these experiments, we determined the relative binding activities of IgG, Fab, and F(ab′)_2_ preparations isolated from the plasmas of otherwise healthy individuals (HS); we also tested a pooled IgG preparation that was commercially purchased. Figs. 1 and 2 present these results, with Fig. 1 showing two HS samples selected to show representative data of IgG and fragment binding to *M. luteus* DNA.

As these data indicate, the Fab fragments from all HS samples failed to bind to *M. luteus* DNA compared with intact IgG. These findings indicate that HS anti-DNA, like SLE anti-DNA, bind by a monogamous bivalent mechanism. Thus, even though the antigenic determinants recognized by HS and SLE DNA are different, the Fab fragments of both types of anti-DNA lack sufficient affinity for monovalent binding.

We next tested the activity of the F(ab′)_2_ fragments. As these data indicate, F(ab′)_2_ fragments from two out of five HS plasmas as well as from the pooled IgG showed similar anti-DNA binding activity compared with that of intact IgG (Figs. 1, 2). Thus, in the Ab response of the individuals tested, the Fc region is not essential.

The interpretation of these experiments depends on the activity of the fragments. To determine whether the Fab and F(ab′)_2_ fragments of HS IgG retained their Ag-binding activity, we tested the relative binding activity of IgG, Fab, and F(ab′)_2_ preparations to EBV Ag and tetanus toxoid. Fab and F(ab′)_2_ fragments prepared from HS plasmas and from the pooled IgG demonstrated levels of binding activity to EBV Ag and tetanus toxoid like those of intact IgG (Fig. 2).

### Binding of IgG, Fab, and F(ab′)_2_ fragments from SLE patients

**FIGURE 3.**
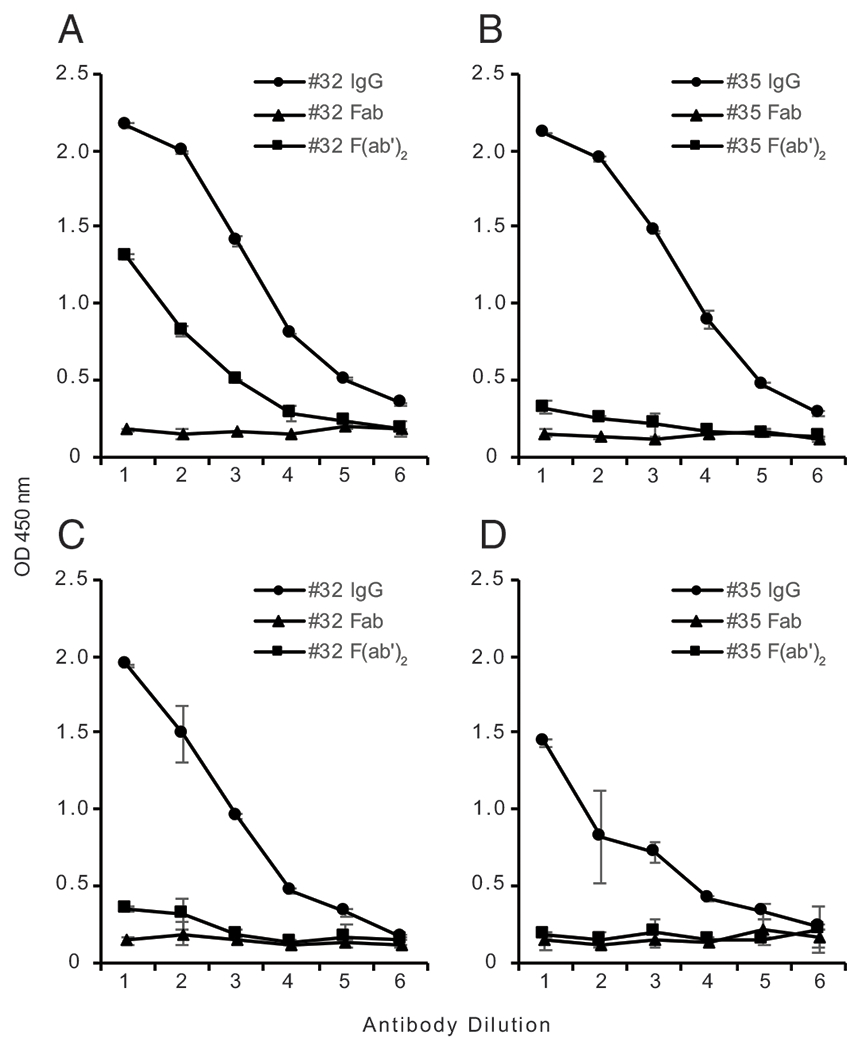
Binding of representative SLE IgG, Fab, and F(ab′)_2_ fragment preparations to *M*. luteus and CT DNA. The binding of IgG (circle), Fab (triangle), and F(ab′)_2_ (square) fragments from two representative SLE plasmas (32 and 35) to *M. luteus* DNA (**A** and **B**) and to CT DNA (**C** and **D**) was examined by ELISA. Each point is the average OD_450_ of two wells, with error bars indicating SD. Serial 2-fold dilutions of IgG and fragments were tested, with initial concentrations as follows. For plasma 32 (**A** and **C**): IgG and F(ab′)_2_, 100 μg/ml, Fab, 62.5 μg/ml. For plasma 35 (**B** and **D**): IgG and F(ab′)_2_, 80 μg/ml, Fab, 50 μg/ml.

**FIGURE 4.**
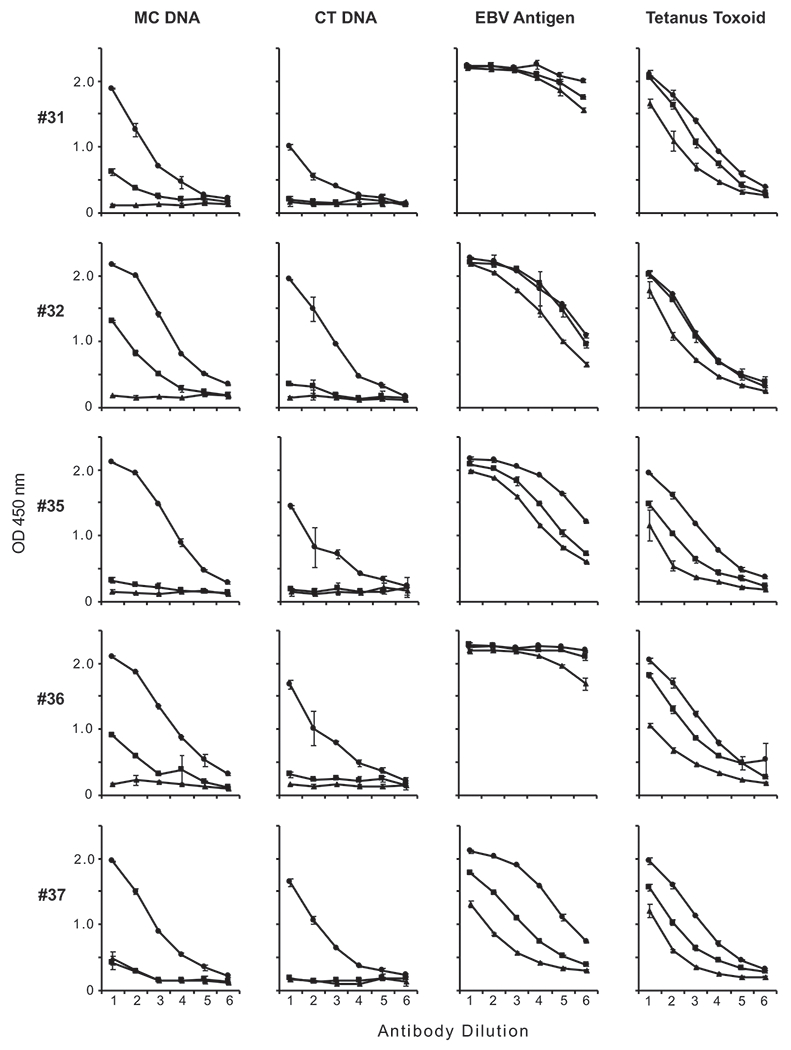
Binding of SLE IgG, Fab, and F(ab′)_2_ fragment preparations to *M*. luteus DNA, CT DNA, and controls. The binding of IgG (circle), Fab (triangle), and F(ab′)_2_ (square) preparations from five SLE plasmas (31, 32, and 35–37) to *M. luteus* DNA, CT DNA, EBV Ag, and tetanus toxoid was examined by ELISA. Each point is the average OD_450_ of two wells, with error bars indicating SD. Serial 2-fold dilutions of IgG and fragments were tested, with initial concentrations as follows. For plasmas 31 and 36: IgG and F(ab′)_2_, 120 μg/ml; Fab, 75 μg/ml. For plasma 32: IgG and F(ab′)_2_, 100 μg/ml; Fab, 62.5 μg/ml. For plasmas 35 and 37: IgG and F(ab′)_2_, 80 μg/ml; Fab, 50 μg/ml.

Because our previous study examined SLE anti-DNA binding to mammalian DNA (i.e., CT DNA) (24), we decided to expand our investigation of anti-DNA in SLE. Specifically, we wanted to determine the binding activity of both Fab and F(ab′)_2_ fragments of SLE IgG with nonconserved determinants on bacterial DNA. Figs. 3 and 4 present these results.

As has been previously shown, Fab and F(ab′)_2_ fragments from all SLE plasmas demonstrated negligible binding activity to CT DNA compared with intact IgG (24). Also consistent with earlier work, Fab and F(ab′)_2_ fragments prepared from SLE sera displayed binding activity to both EBV and tetanus toxoid. These results confirm and extend our previous findings on the role of Fc-dependent monogamous bivalency, with all SLE plasmas tested thus far displaying this binding mechanism.

Having confirmed our previous results on autoantibodies to DNA, we then investigated the binding of fragments from SLE plasmas to *M. luteus* DNA. Prior studies have indicated that blood from some patients with SLE contains at least two populations of anti-DNA (32). Whereas one population (i.e., autoantibodies) binds to conserved sites on mammalian and bacterial DNA, another population binds specifically to bacterial DNA; specific binding to bacterial DNA suggests retention of the ordinary mode of binding to nonconserved DNA sites. We therefore wanted to test whether SLE plasma contains Abs to *M. luteus* DNA and whether the binding of these Abs requires the Fc region.

As shown in Figs. 3 and 4, we found that Fab fragments from SLE plasmas had negligible binding activity to *M. luteus* DNA similar to that observed with HS fragments. By contrast, F(ab′)_2_ fragments from two out of five SLE plasmas showed significant binding activity to *M. luteus* DNA, albeit lower than that of intact IgG. Together, these findings indicate that, whereas the autoantibody response to DNA of SLE IgG requires the presence of the Fc region, the binding to bacterial DNA does not have this requirement whether in HS or SLE patients.

## DISCUSSION

The results of these experiments, to our knowledge, provide new insight into the mechanisms of anti-DNA binding in normal as well as aberrant immunity. As shown in previous studies, autoantibodies to DNA in patients with SLE interact with DNA by a binding mode termed monogamous bivalency. In this mode, both Fab sites contact antigenic determinants on the same extended polynucleotide chain (24, 26). The necessity for bivalency reflects the low affinity of each Fab site for DNA. Whereas prior work established monogamous bivalent binding of autoantibodies to DNA in SLE, this study demonstrates that Abs to bacterial DNA in otherwise healthy individuals also use this mechanism.

The necessity for monogamous bivalency in HS anti-DNA is perhaps surprising because these Abs arise in otherwise healthy individuals, likely in response to foreign DNA from bacterial infection or colonization. HS anti-DNA bind to nonconserved sites on foreign DNA and might be expected to have high affinity comparable with that of Abs to foreign proteins. Nevertheless, as our experiments demonstrated, Fab fragments of HS IgG failed to bind to *M. luteus* DNA under the assay conditions used. This finding suggests that there are limitations in the generation of high-affinity anti-DNA, whether in HS in their response to foreign DNA or in patients with SLE in their response to self-DNA.

The lack of Abs capable of monovalent interaction suggests restrictions in Ag selection whether in normal or aberrant immunity. Perhaps the preimmune repertoire lacks precursors that can be mutated to form a high-affinity binding site capable of a monovalent interaction. An alternative explanation for the pattern of binding observed may relate to the properties of DNA as an immunogen, with susceptibility to DNase digestion limiting the amount of DNA that can contact surface receptors on B cells to drive an Ag-specific response (33, 34). Based on studies of cfDNA, circulating DNA from bacterial sources has a short half-life in blood as well as a low molecular mass (35, 36). In this regard, the pathways of B cell activation may affect the avidity of anti-DNA induced. Of note, studies in murine infection and lupus models have shown that pathogenic autoantibodies may develop by T cell–dependent germinal center reactions as well as extrafollicular reactions (37, 38).

In our previous work, we showed that SLE autoantibodies to DNA display a unique type of bivalent interaction that requires the presence of the Fc region (24). Even though the F(ab′)_2_ fragments of IgG anti-DNA are capable of bivalent binding, in our prior studies, the fragments failed to bind to CT DNA. Control studies showed that the F(ab′)_2_ fragments, as well as Fab fragments, were functional because they bound both tetanus toxoid and an EBV Ag preparation. We thus termed this observed binding mechanism Fc-dependent monogamous bivalency. Our prior study involved samples from five patients. We have now observed similar results in samples from another five patients with SLE to bring the total number of samples to 10, strongly suggesting that Fc-dependent monogamous bivalency is the usual binding mode for autoantibodies to DNA.

The impact of Fc on Ag binding has been previously characterized in studies on the binding of murine monoclonal Abs to fungal capsular polysaccharides, bacterial polysaccharides, and DNA (29–31, 39, 40). Rather than using fragments produced by enzyme digestion, these studies characterized the binding activity of molecular constructs in which the same variable regions were joined with H chains of different isotypes. Importantly, these constructs varied in their strength and specificity of Ag binding, pointing to some type of structural connection or communication from the Fc region to the variable regions. For anti-DNA Abs, H chain isotype also influenced cross-reactivity with laminin and collagen IV as well as pathogenicity (41). Furthermore, computer-generated models and spectroscopy of Ab–Ag complexes in solution have indicated that sequence changes to the Fc region influence the structures of Ab–Ag complexes (41, 42).

In addition to induced allosteric changes in Ab structure, the Fc region may also influence Ab binding through other means, such as Fc–Fc interactions. Greenspan and colleagues (43) showed that murine Abs to streptococcal polysaccharides can bind cooperatively, with the expression of IgG3 in particular enhancing binding avidity. Crystal structures of IgG3 in solution indicate that the single disulfide bond in the hinge region of IgG3 confers greater flexibility and allows for increased interaction with other IgG molecules compared with other isotypes (44). The Fc dependence of anti-DNA in SLE suggests that autoantibodies to DNA have low binding avidity without Fc–Fc interactions, with cooperativity needed to increase binding activity. A direct interaction of Fc with DNA is also possible, creating an additional binding site. In preliminary experiments (data not shown), however, we did not observe binding of Fc fragments to *M. luteus* DNA under the conditions of the ELISA.

We have thus far studied only a limited number of both HS and SLE preparations because of availability of samples in adequate amounts to prepare fragments, which is labor intensive and requires sufficient titers of anti-DNA. Although these studies can be considered as pilot experiments, we nevertheless showed that F(ab′)_2_ fragments from the blood of both groups can bind *M. luteus* DNA. F(ab′)_2_ fragments prepared from the pooled IgG, which we presume came from many individuals, also demonstrated significant binding activity to *M. luteus* DNA. In this property, the SLE samples resembled those from HS, suggesting that the binding to nonconserved sites on foreign DNA differs from that of SLE anti-DNA, for which a fully intact IgG structure is required for binding to self-DNA.

In these experiments, we have used an ELISA, which is a solid phase assay that potentially could affect the antigenicity of DNA. In previous experiments, we compared the antigenicity of DNA either coated directly to plates with that of biotinylated DNA bound to the solid phase via streptavidin; binding of biotinylated DNA to streptavidin assures that the DNA is soluble and freely mobile in the fluid phase (24). In those experiments, we did not observe differences with respect to the activity of the Fab and F(ab′)_2_ fragments. Both were inactive in the conventional ELISA as well as the “sandwich” ELISA with streptavidin. We have hypothesized that, with high molecular mass DNA as we used in the current experiments, much of the DNA is effectively in the fluid phase and capable of any rearrangements or topologic changes needed for antigenicity. In contrast, with low molecular mass DNA, DNA is likely more adherent to the solid phase, and interaction with Ab can be affected (45).

As demonstrated previously by adsorption experiments, anti-DNA in patients with SLE are mixtures of autoantibodies to conserved sites (i.e., DNA backbone) on all DNA as well as Abs to nonconserved sites on foreign DNA (32). In the current study, the binding of F(ab′)_2_ fragments to *M. luteus*, but not CT, DNA provides further evidence for the presence of Abs to foreign DNA in the blood of SLE patients.

The differences in the role of Fc dependence on the interaction of anti-DNA with self-DNA and foreign DNA have implications for the mechanisms of DNA Ag selection. An important distinction between mammalian and bacterial DNA relates to their immunological properties. Whereas mammalian DNA is immunologically inactive if not inhibitory (1), bacterial DNA is immunologically active by virtue of its CpG motifs (46–49). These motifs can serve as adjuvants (50), and in vitro studies have demonstrated that CpG oligonucleotides can promote native B cell progression to Ab-secreting plasma cells without requiring BCR cross-linking (51). The presence of this adjuvant activity may enable the generation of higher affinity IgG Abs than would occur with mammalian DNA even in the setting of autoimmunity. Because of their increased affinity, Abs to foreign DNA, although binding by monogamous bivalent interactions, may nevertheless not require the presence of the Fc region for stable interaction.

Together, our findings suggest that anti-DNA Abs bind by monogamous bivalency to both foreign and self-DNA; the response to foreign DNA in both SLE patients and HS, however, does not invariably require the Fc region. This study also provides further evidence that patients with SLE can produce different types of anti-DNA: variably Fc-dependent Abs to foreign DNA like those found in otherwise healthy individuals and consistently Fc-dependent Abs to self-DNA. Future studies will define further the binding properties of these two types of anti-DNA and delineate in greater detail how the presence of the Fc region influences anti-DNA binding.

## Abbreviations used in this article:

anti-DNA: Ab to DNA
cfDNA: cell-free DNA
CT: calf thymus
HS: healthy subject
SLE: systemic lupus erythematosus
